# Prevalence of Adverse Pregnancy Outcomes in Women With and Without Gestational Diabetes Mellitus in Al-Baha Region, Saudi Arabia

**DOI:** 10.7759/cureus.52421

**Published:** 2024-01-17

**Authors:** Tajelsir Osman, Eman A Keshk, Meshari A Alghamdi, Faisal A Alzahrani, Abdulhakim Abdulrazaq M Alghamdi, Ayman G Alzahrani, Yahya Alzahrani, Mohammed Ahmed A Alghamdi, Adnan Saleh I Alghamdi, Abdulelah Abdulrazaq M Alghamdi

**Affiliations:** 1 Obstetrics & Gynecology, Al-Baha University, Al-Baha, SAU; 2 College of Medicine, Al-Baha University, Al-Baha, SAU; 3 Clinical Pharmacy, Al-Baha University, Al-Baha, SAU

**Keywords:** glucose intolerance in pregnancy, gdm therapy and monitoring, pregnancy-related complications, pregnancy complications, saudi arabia, al-baha region, prevalence, outcome of gestational diabetes

## Abstract

Background: Gestational diabetes mellitus (GDM) is a condition characterized by glucose intolerance that develops during pregnancy. It is associated with adverse maternal and fetal outcomes and has long-term health implications for both the mother and the child. This study aimed to estimate the prevalence of adverse pregnancy outcomes in women with and without GDM in the Al-Baha region, Saudi Arabia.

Methods: A cross-sectional study was conducted in the Al-Baha region from April 2023 to November 2023. The study included mothers residing in the Al-Baha region who were willing to participate and had access to a social media account. A simple random sampling technique was used, and the estimated sample size was 422. A self-administered electronic questionnaire was used to collect data on socio-demographic and lifestyle factors, as well as the pregnancy outcomes of diabetic and non-diabetic mothers. Descriptive and inferential statistical analyses were performed using IBM SPSS Statistics for Windows, Version 28.0 (Released 2012; IBM Corp., Armonk, New York, United States).

Results: We included 422 women in the study with the majority of participants in the age group of 36-40 years(15.4%, n=74). Most participants (66.6%, n=321) had attained a university degree, and a significant proportion resided in Al-Baha City (52.3%, n=252). Maternal outcomes indicated a significant association between GDM and the development of eclampsia (OR = 8.296, 95%CI: 4.353-15.810, p < 0.001), as well as an increased risk of thyroid diseases (OR = 2.723, 95%CI: 1.428-5.193, p = 0.002). Fetal outcomes revealed a significant association between GDM and respiratory distress/lack of oxygen in newborns (OR = 2.032, 95%CI: 1.085-3.805, p = 0.024), and infants of GDM patients had a higher risk of hypoglycemia (OR = 8.099, 95%CI: 3.350-19.581, p < 0.001).

Conclusion: We found that GDM increased the risk of complications such as eclampsia, thyroid problems, and postpartum hemorrhage. GDM was also associated with shorter pregnancy durations, higher cesarean section rates, and an increased risk of developing type 2 diabetes post pregnancy. The study emphasized the importance of comprehensive GDM therapy and monitoring.

## Introduction

Gestational diabetes mellitus (GDM) is defined as glucose intolerance that occurs and is first diagnosed during pregnancy, whereas pre-gestational diabetes is defined as the presence of diabetes mellitus (DM) before pregnancy [1,2]. The incidence of diabetes has been on the rise globally, and it is noteworthy that approximately 16.6% of pregnant women are affected by hyperglycemia. Among this group, a significant majority, accounting for 84%, are diagnosed with GDM [1,3].

Hyperglycemia during pregnancy is associated with various maternal complications, including pre-eclampsia. It can also lead to fetal complications such as macrosomia, shoulder dystocia, an increased risk of stillbirth, respiratory distress, hypoglycemia, and neonatal hypoglycemia [4-6]. Long-term effects of GDM on mothers and their children have also been seen, including an increased risk of developing type 2 DM (T2DM), maternal and childhood obesity, and cardiovascular disease [7]. GDM occurs as a result of pancreatic function insufficient to overcome the insulin resistance associated with the pregnant state. Control of blood glucose levels during pregnancy reduces morbidity for both mother and baby [8]. A previous study in Saudi Arabia showed that the prevalence of GDM was 13.8%, and T2DM was 0.9%. The T2DM group had the highest mean parity and shortest mean gestational age as compared to other groups. Half of all the subjects in the T2DM group also experienced preterm labor, as opposed to only 10% in GDM and 14% in the non-DM group, respectively. Finally, neonates delivered by T2DM mothers had the highest percentage of admissions to the neonatal intensive care unit (NICU) (33%) as compared to 10% in the non-DM group and only 5% in the GDM group [9]. Another study in Mulago Hospital, Uganda showed about 50% of women with GDM were obese [10]. So, we conducted this study to estimate the prevalence of adverse pregnancy outcomes in women with and without GDM in the Al-Baha region, Saudi Arabia.

## Materials and methods

This cross-sectional study was conducted in the Al-Baha region, Saudi Arabia, from April 1, 2023, to November 30, 2023. The target population consisted of women residing in the Al-Baha region of Saudi Arabia who were willing to participate in the study. The inclusion criteria for the study required participants to be mothers residing in the Al-Baha Region, regardless of their nationality. On the other hand, exclusion criteria were implemented to maintain the integrity and relevance of the study. Women who did not reside in the Al-Baha region and individuals who declined to participate or share their information for the study were excluded.

Sampling technique and sample size

Participants were included using a simple random sample procedure. The sample size was computed using the Raosoft online sample size calculator. Based on a 95% confidence interval, 5% margin of error, the estimated sample size was 384, and we adjusted it to 422 to compensate for the 10% non-response rate.

Data collection

A self-administered electronic questionnaire was sent online to determine the prevalence of adverse pregnancy outcomes in women with and without GDM. The questionnaire was developed based on a comprehensive review of previous literature related to GDM and adverse pregnancy outcomes [11,12]. The questionnaire was designed to capture relevant information and insights from participants. To ensure its validity and reliability, it was subjected to a rigorous review process. The questionnaire underwent external review by two independent experts in the field of obstetrics and gynecology, who were not involved in the research as authors or participants. The questionnaire consisted of two sections: (i) socio-demographic and lifestyle data and (ii) the outcome of pregnancy (in diabetic and non-diabetic mothers).

Data analysis

The data were entered into Microsoft Excel (Microsoft Corporation, Redmond, Washington, United States) for cleaning and analyzed with IBM SPSS Statistics for Windows, Version 28.0 (Released 2012; IBM Corp., Armonk, New York, United States). Both descriptive and inferential statistics were used to examine the relationship between maternal and neonatal factors in both GDM and non-GDM patients. Descriptive statistics were used to summarize the characteristics of the study population, while inferential statistics, such as chi-square tests and Fisher's exact tests, were employed to assess the association between these factors and the presence of GDM. Additionally, multiple regression analysis was conducted to further assess the associations between the factors and calculate odds ratios. The results were presented in tables, providing valuable insights into the relationship between maternal and neonatal factors and the occurrence of GDM.

Ethical consideration

The study was approved by the Al-Baha University Research Committee (approval number: REC/OB/BU-FM/2023/14). Measures were taken to ensure the confidentiality of participants' personal information, such as using anonymized identifiers instead of personal identifiers in the data analysis and reporting. Only authorized personnel involved in the study had access to the data, and data storage systems were secured with appropriate safeguards to prevent unauthorized access or data breaches. 

## Results

Sociodemographic data

The findings revealed that 14.5% of participants were in the age group of 18-25 years, followed by 26-30 years (13.7%), 31-35 years (13.5%), and 36-40 years (15.4%). A small proportion (5%) had completed primary to intermediate education, while the majority (66.6%) had attained a university degree or equivalent. High school graduates constituted 23.2% of the sample, and postgraduates accounted for 5.2%. In terms of employment status, the largest proportion of participants were housewives (61.6%). Finally, most of the participants resided in Al-Baha city (52.3%), followed by Beljurashi (25.1%), with other cities representing smaller proportions (Table 1).

**Table 1:** Sociodemographic data Data has been given as counts and percentages.

Sociodemographic Data		Count (n)	Percentage (%)
Age Grouping	18-25 Years	70	14.5%
	26-30 Years	66	13.7%
	31-35 Years	65	13.5%
	36-40 Years	74	15.4%
	41-45 Years	73	15.1%
	45-50 Years	69	14.3%
	51-55 Years	65	13.5%
Educational status	Primary - Intermediate	24	5.0%
	High School	112	23.2%
	University or equivalent	321	66.6%
	Postgraduate	25	5.2%
Employment status	Housewife	297	61.6%
	Work within the health sector	29	6.0%
	Work outside the health sector	156	32.4%
City of residence	Al-Baha	252	52.3%
	Bani-Hassan	18	3.7%
	Beljurashi	121	25.1%
	Al-Aqiq	0	0.0%
	Al-Qaraa	13	2.7%
	Al-Makhwa	11	2.3%
	Ghamid-Alzinad	15	3.1%
	Al-Mandaq	30	6.2%
	Qelwa	22	4.6%

Pre-pregnancy diabetes and GDM

It was found that a notable proportion (21.0%) had a previous diabetes diagnosis. Among those with a previous diabetes diagnosis, 33.7% had type 1 DM (T1DM) and 66.3% had T2DM. Furthermore, 23.4% reported a diagnosis of GDM. Additionally, the average number of pregnancies reported by the participants was 4±2 (Table 2).

**Table 2:** Pre-pregnancy diabetes and gestational diabetes assessment Data has been given as counts and percentages except for the times of pregnancy, which is represented as Mean and SD.

		Count (n)		Percentage (%)	
Were you diagnosed with diabetes before pregnancy?	No	381		79.0%	
	Yes	101		21.0%	
If the answer to the previous question is yes: What is the type of diabetes?	T1DM	34		33.7%	
	T2DM	67		66.3%	
Have you been diagnosed with gestational diabetes?	No	369		76.6%	
	Yes	113		23.4%	
How many times have you been pregnant?		Mean	3.8	SD	2.2

Maternal and neonatal factors in GDM and non-GDM patients

The result found that non-GDM participants had a higher proportion of individuals weighing between 50-70 kg (58.5%) compared to those with GDM (45.1%) (p = 0.007). A higher percentage of participants in the non-GDM group had a natural delivery (71.5%) compared to the GDM group (54.0%), where a higher proportion underwent a cesarean section (46.0%) (p < 0.001). The gender distribution of babies was similar between the two groups, with males comprising the majority (54.7% in non-GDM and 60.2% in GDM) (p = 0.309). Regarding baby weight, a higher percentage of babies in the GDM group weighed 3.5-4 kg (16.1%) compared to the non-GDM group (5.1%) (p < 0.001) (Table 3).

**Table 3:** Maternal and neonatal factors in GDM and non-GDM patients ^a^ Significant difference, which is p<0.05; ^b^ Fisher's exact test GDM: gestational diabetes mellitus

		Non-GDM (n=369)		GDM (n=113)		P-value
		Count (n)	Percentage (%)	Count (n)	Percentage (%)	
Weight of mother during pregnancy (kg)	Less than 50	37	10.0%	7	6.2%	0.007^a^
	50-70	216	58.5%	51	45.1%	
	70-90	100	27.1%	45	39.8%	
	More than 90	16	4.3%	10	8.8%	
Duration of the pregnancy	Less than 37 weeks	75	20.3%	22	19.5%	0.029^ a^
	37 to 42 weeks	228	61.8%	82	72.6%	
	More than 42 weeks	66	17.9%	9	8.0%	
Mode of delivery	Natural (Vaginal)	264	71.5%	61	54.0%	<0.001^ a^
	Cesarean	105	28.5%	52	46.0%	
Gender of the baby	Male	202	54.7%	68	60.2%	0.309
	Female	167	45.3%	45	39.8%	
Weight of baby at birth	Less than 1 kg	11	3.0%	2	1.8%	<0.001^ a, b^
	1-1.5 kg	31	8.4%	7	6.3%	
	1.6-2 kg	49	13.3%	14	12.5%	
	2-2.5 kg	93	25.2%	29	25.9%	
	2.5-3 kg	96	26.0%	22	19.6%	
	3-3.5 kg	63	17.1%	13	11.6%	
	3.5-4 kg	19	5.1%	18	16.1%	
	4-4.5 kg	7	1.9%	6	5.4%	
	More than 5 kg	0	0%	1	0.9%	

Comparison of obstetric complications and postpartum health in GDM and non-GDM patients

Based on our research comparing obstetric complications and postpartum health between patients with GDM and those without (non-GDM), significant differences were observed. GDM patients had a higher incidence of high blood pressure during pregnancy (eclampsia) (27.4% vs. 4.6%, p < 0.001) and thyroid disease during pregnancy (15.9% vs. 7.6%, p < 0.001) compared to non-GDM patients. Additionally, GDM patients had a higher prevalence of bleeding after giving birth (25.7% vs. 8.1%, p < 0.001) and a greater likelihood of developing T2DM after pregnancy (27.4% vs. 4.3%, p < 0.001) (Table 4). 

**Table 4:** Comparison of obstetric complications and postpartum health in GDM and non-GDM patients ^a^ Significant difference which is p<0.05; ^b^ Fisher's exact test GDM: gestational diabetes mellitus

		Non-GDM (n=369)		GDM (n=113)		P-value
		Count (n)	Percentage (%)	Count (n)	Percentage (%)	
Did you develop high blood pressure during pregnancy (eclampsia)?	No	323	87.5%	71	62.8%	<0.001^a^
	Yes	17	4.6%	31	27.4%	
	I don't know	29	7.9%	11	9.7%	
Did you suffer from thyroid disease during pregnancy?	No	305	82.7%	72	63.7%	<0.001^a^
	Yes	28	7.6%	18	15.9%	
	I don't know	36	9.8%	23	20.4%	
If the answer is yes to the previous question, did you get	Hypothyroidism	19	67.9%	13	72.2%	1.000^ b^
	Hyperthyroidism	6	21.4%	4	22.2%	
	I don't know	3	10.7%	1	5.6%	
Did you experience bleeding during pregnancy?	No	315	85.4%	89	78.8%	0.245
	Yes	35	9.5%	16	14.2%	
	I don't know	19	5.1%	8	7.1%	
Did you have bleeding after giving birth?	No	324	87.8%	76	67.3%	<0.001^a ^
	Yes	30	8.1%	29	25.7%	
	I don't know	15	4.1%	8	7.1%	
Did you get vaginal infections during pregnancy (vaginal thrush)?	No	254	68.8%	67	59.3%	0.159
	Yes	88	23.8%	34	30.1%	
	I don't know	27	7.3%	12	10.6%	
Did you develop type 2 diabetes after pregnancy?	No	330	89.4%	68	60.2%	<0.001^ a^
	Yes	16	4.3%	31	27.4%	
	I don't know	23	6.2%	14	12.4%	

Several significant differences were observed in obstetric complications and postpartum health. GDM patients had a higher incidence of increased amniotic fluid around the fetus (15.9% vs. 4.1%, p < 0.001), dystocia (43.4% vs. 19.0%, p < 0.001), and NICU admission (14.2% vs. 9.8%, p = 0.012) compared to non-GDM patients. Additionally, GDM patients had a higher prevalence of hypoglycemia in newborns (14.2% vs. 2.2%, p < 0.001) (Table 5). 

**Table 5:** Comparison of obstetric complications and postpartum health in GDM and non-GDM patients ^a^ Significant difference which is p<0.05; ^b ^Fisher-Exact test GDM: gestational diabetes mellitus

		Non-GDM (n=369)		GDM (n=113)		P-value
		Count (n)	Percentage (%)	Count (n)	Percentage (%)	
Was the fetus aborted?	No	334	90.5%	95	84.1%	0.055
	Yes	35	9.5%	18	15.9%	
Increased amniotic fluid around the fetus?	No	255	69.1%	62	54.9%	<0.001^ a^
	Yes	15	4.1%	18	15.9%	
	I don't know	99	26.8%	33	29.2%	
Dystocia?	No	299	81.0%	58	51.3%	<0.001 ^a,b^
	Yes	70	19.0%	49	43.4%	
	I don't know	0	0.0%	6	5.3%	
Did the newborn cry immediately after birth?	No	33	8.9%	16	14.2%	0.263
	Yes	258	69.9%	73	64.6%	
	I don't know	78	21.1%	24	21.2%	
Did the baby suffer from convulsions after birth?	No	320	86.7%	94	83.2%	0.089
	Yes	6	1.6%	6	5.3%	
	I don't know	43	11.7%	13	11.5%	
Was the baby admitted to neonatal intensive care?	No	313	84.8%	83	73.5%	0.012^ a^
	Yes	36	9.8%	16	14.2%	
	I don't know	20	5.4%	14	12.4%	
Reason of admission to neonatal intensive care?	Lack of oxygen	16	44.4%	7	43.8%	-
	jaundice	11	30.6%	4	25.0%	
	Low Birth Weight	3	8.3%	2	12.5%	
	Hypoglycemia	2	5.6%	3	18.8%	
	Infection	3	8.3%	0	0.0%	
	High Birth Weight	1	2.8%	0	0.0%	
Did the newborn suffer from jaundice (yellowing of the skin)?	No	209	56.6%	61	54.0%	0.881
	Yes	136	36.9%	44	38.9%	
	I don't know	24	6.5%	8	7.1%	
Did the newborn suffer from respiratory distress/lack of oxygen after birth?	No	308	83.5%	88	77.9%	0.062
	Yes	31	8.4%	18	15.9%	
	I don't know	30	8.1%	7	6.2%	
Did the newborn suffer from hypoglycemia?	No	328	88.9%	81	71.7%	<0.001^ a^
	Yes	8	2.2%	16	14.2%	
	I don't know	33	8.9%	16	14.2%	

Comparative analysis of maternal and fetal outcomes in GDM and non-GDM patients

In the comparison between patients with GDM with those without (non-GDM), several significant associations were identified. Maternal outcomes showed a significant association between GDM and the development of eclampsia (OR = 8.296, 95%CI: 4.353-15.810, p < 0.001) as well as an increased risk of thyroid diseases (OR = 2.723, 95%CI: 1.428-5.193, p = 0.002). Although there was no significant association with bleeding during pregnancy (p = 0.136), GDM patients had a higher risk of bleeding after giving birth (OR = 4.121, 95%CI: 2.335-7.274, p < 0.001). Vaginal infections during pregnancy did not show a significant association (p = 0.117), but GDM patients had a significantly higher risk of developing T2DM after pregnancy (OR = 9.403, 95%CI: 4.873-18.144, p < 0.001). Fetal outcomes revealed a significant association between GDM and increased amniotic fluid (OR = 4.935, 95%CI: 2.356-10.337, p < 0.001), as well as a higher risk of dystocia (OR = 3.609, 95%CI: 2.276-5.721, p < 0.001). While there was no significant association with the immediate cry of the newborn (p = 0.102), GDM patients had an increased risk of convulsions after birth (OR = 3.404, 95%CI: 1.073-10.802, p = 0.028). The admission to NICU did not show a significant association (p = 0.109), and there was no significant association between GDM and jaundice in newborns (p = 0.649). However, respiratory distress/lack of oxygen in newborns was significantly associated with GDM (OR = 2.032, 95%CI: 1.085-3.805, p = 0.024), and GDM neonates had a higher risk of hypoglycemia (OR = 8.099, 95%CI: 3.350-19.581, p < 0.001) (Table 6). 

**Table 6:** Comparative analysis of maternal and fetal outcomes in GDM and non-GDM patients: significant associations and odds ratios Significant difference represented when p<0.05

Maternal Outcome Variable	P-value	Odd ratio	95% CI (Lower)	95% CI (Upper)
Eclampsia	<0.001	8.296	4.353	15.810
Thyroid diseases	0.002	2.723	1.428	5.193
Bleeding during pregnancy	0.136	1.618	.856	3.058
Bleeding after giving birth	<0.001	4.121	2.335	7.274
Vaginal infections during pregnancy (vaginal thrush)	0.117	1.465	.908	2.364
Type 2 diabetes after pregnancy	<0.001	9.403	4.873	18.144
Fetal Outcome Variable				
Fetal abortion	0.055	1.808	.980	3.336
Increased amniotic fluid around the fetus	<0.001	4.935	2.356	10.337
Dystocia	<0.001	3.609	2.276	5.721
Newborn cry immediately after birth	0.102	.584	.304	1.119
Suffer from convulsions after birth	0.028	3.404	1.073	10.802
Admitted to neonatal intensive care	0.109	1.676	.887	3.168
Jaundice	0.649	1.108	.711	1.728
Respiratory distress/lack of oxygen	0.024	2.032	1.085	3.805
Hypoglycemia	<0.001	8.099	3.350	19.581

## Discussion

This study produced important information on unfavorable pregnancy outcomes in women with and without GDM. The important results from Tables 3-6 are discussed below to help evaluate the study. To support the findings, the discussion compares pertinent research from the literature. The discussion also discusses the results' larger ramifications, highlighting the current study's importance to maternal and fetal health in the area.

Table 3 indicates how GDM patients vary from non-GDM patients in maternal and neonatal parameters. A key finding is the pregnancy duration gap. GDM patients had shorter pregnancies (19.5% < 37 weeks) compared to non-GDM patients (20.3% < 37 weeks) (p < 0.029). GDM patients must be closely monitored to maximize pregnancy length and newborn health. In addition, 46.0% of GDM patients choose cesarean section births, compared to 28.5% in the non-GDM group (p < 0.001). The delivery method affects maternal and newborn health. This highlights the need for seamless healthcare provider coordination to overcome this discrepancy and guarantee safer mother-baby births. Global studies such as Athukorala et al.'s 2010 study show that obese women are more likely to have cesarean sections, following GDM patterns [13]. This is also consistent with Negrato et al. [14]. GDM patients had a higher risk of shorter pregnancy durations, which supports Feng et al.'s study and emphasizes the necessity for careful treatment [15]. These findings highlight the need to know the relationship between GDM, shorter pregnancies, and more cesarean section deliveries. These risk factors must be recognized, GDM patients constantly monitored, and treatments devised to maximize pregnancy length and delivery approaches to provide the best outcomes for mothers and infants.

Tables 4-5 show the complex landscape of obstetric problems and postpartum health in GDM patients compared to non-GDM patients. These tables reveal stark differences with major ramifications. First, p-values lower than 001 show that GDM patients have a greater risk of eclampsia and thyroid disorders during pregnancy. In 2012, Bodmer-Roy and colleagues stressed the need for early identification and watchful treatment of these problems in GDM pregnancies [12]. Prevention is key for mother-child health. GDM patients had a greater risk of postpartum hemorrhage (p-value <0.001), underscoring the need for customized therapy. Furthermore, in line with Shams et al., healthcare providers prioritize addressing the potentially hazardous complications associated with GDM [16]. GDM patients had a significantly greater chance of acquiring T2DM during pregnancy (p <0.001). Slowing this chronic condition and protecting the mother's health requires long-term monitoring and help. GDM patients had similar risks of eclampsia, thyroid problems, postpartum hemorrhage, and T2DM, according to Bodmer-Roy et al. and Schmidt et al. [12,17]. These consistent findings across studies support GDM awareness, prevention, and therapy throughout pregnancy and beyond.

GDM and non-GDM maternal and fetal outcomes are compared in Table 6. The findings show that women with GDM suffer significant risks, stressing the necessity for specialist treatment and surveillance throughout pregnancy and the postpartum period. The increased incidence of eclampsia and thyroid disorders in GDM patients was worrying. Women with GDM had an 8.40-fold higher risk of life-threatening eclampsia. Thyroid disorders during pregnancy were also more common in GDM patients. Bodmer-Roy et al. similarly found that GDM patients had 4.12 times the risk of postpartum hemorrhage, emphasizing the necessity for careful monitoring and treatment [12]. The findings stressed the significance of postpartum GDM assistance. Long-term GDM health effects were a major finding. GDM women are 9.4 times more likely to acquire T2DM after pregnancy, highlighting the necessity for continued health care. Malaza et al. estimated that GDM accounts for a considerable fraction of maternal diabetes cases worldwide [18]. GDM patients had more amniotic fluid and dystocia, requiring extra care. These findings complement Athukorala et al. [13] and point to comprehensive treatment. GDM mothers' newborns risk respiratory discomfort and hypoglycemia. This suggests newborn health concerns. Feng et al. found that GDM pregnancies consistently result in respiratory distress [15]. Lin et al. found a risk of newborn hypoglycemia, emphasizing the need for postnatal care for GDM neonates [19]. This shows that pregnant women with GDM suffer increased risks and their effects on maternal and fetal outcomes. The findings confirm prior studies and underscore the need for personalized GDM therapy, monitoring, and postnatal assistance. These concerns must be recognized to improve GDM moms' and babies' health.

There is thus a need for comprehensive GDM therapy. Healthcare professionals must monitor eclampsia and thyroid disorders during and after pregnancy. Early intervention may enhance mother-infant outcomes. GDM patients' high risk of postpartum hemorrhage underscores the need for prolonged postnatal care and surveillance. Women with GDM are more vulnerable, so they need help and resources throughout this vital recovery and transition period. GDM patients' high risk of T2DM after pregnancy requires continuing monitoring and treatment. Such individuals should get lifestyle advice and frequent checkups to reduce long-term health risks. Finally, the data emphasize the need for thorough and ongoing GDM treatment. Recognizing and treating these hazards during pregnancy and beyond is crucial to maternal and child health.

Limitations

This research has some limitations to consider before evaluating its conclusions. Sampling bias is a major issue. The study's sample, mostly highly educated women, may not fully reflect Al-Baha's population [20]. This bias may restrict the results' external validity, making it difficult to generalize them to a wider demographic. Secondly, self-reported data may be limited. Recall bias might affect self-reporting because individuals may misremember their health or medical history. This dependence on memory and perception may affect data accuracy and dependability. The study's cross-sectional design is the third restriction [20]. This sort of inquiry may find connections, but not causality. Longitudinal or cohort studies are needed to identify GDM risk factor causal connections [20]. Lastly, the study lacks clinical data that might improve GDM risk factor knowledge. Participants' BMI, nutrition, and family medical history may reveal GDM causes. Without these characteristics, the condition cannot be can't fully comprehended. Overall, sampling bias, self-reported data, a cross-sectional design, and a lack of clinical information restrict the research. Researchers and readers should recognize these limitations when evaluating the data and their implications for the Al-Baha region.

Recommendations

GDM screening must be regular in prenatal care. Early GDM risk detection allows for prompt interventions and individualized care [21]. Also, comprehensive health education should empower healthcare practitioners and pregnant women. These initiatives should raise awareness regarding GDM risk factors, preventative, and management [22,23]. Healthcare practitioners must keep current on GDM standards and research, while pregnant women should be informed about healthy living, diet, and prenatal checkups to reduce GDM risks. Furthermore, emphasizing frequent prenatal checkups, particularly for GDM patients, is crucial. These checkups should assess blood sugar, blood pressure, and other health factors [24]. Early diagnosis and treatment of problems may improve maternal and newborn outcomes, saving healthcare costs and enhancing care.

Comprehensive longitudinal studies are needed to understand GDM and its effects. Such research may delve into investigating causality and the several causes of GDM in Al-Baha, similar to previous studies conducted by Popova et al. in 2023 [24] [24]. By improving scientific knowledge of GDM in this environment, local treatments and tactics may be tailored to be most successful. The Al-Baha healthcare system may improve maternal and newborn health outcomes for GDM patients by following these suggestions and expanding on the study's results. This comprehensive strategy, including screening, education, monitoring, and research, may significantly reduce GDM-related poor pregnancy outcomes and enhance regional healthcare.

## Conclusions

GDM increases the risk of eclampsia, thyroid problems, and postpartum hemorrhage. Comprehensive GDM therapy and surveillance from early pregnancy to the postpartum period may benefit mothers and babies. GDM patients are more likely to acquire T2DM following pregnancy, according to the research. The need for continued postpartum care and glucose monitoring in GDM patients is highlighted. The study also identified a strong association between GDM and shorter pregnancy durations and higher cesarean section rates, underscoring the necessity for joint healthcare treatments to maximize pregnancy length and delivery modalities in GDM patients. The study highlights the need for early discovery, appropriate treatment, and continued care to enhance maternal and newborn outcomes and long-term health in GDM. Integrating these results into clinical practice may improve GDM-affected pregnancy care for mothers and newborns.

## Appendices

Questionnaire

**Table 7:** Questionnaire

18_25 years.	The age
26_30 years.	
31_35 years.	
36_40 years.	
41_45 years.	
46_50 years.	
51_55 years.	
Elementary	Educational level
Intermediate	
High school certificate	
A university degree or its equal	
Postgraduate certificate	
Housewife.	Where do you work?
work in the health sector.	
work outside the health sector.	
Al Baha.	The area where you live.
Baljurashi.	
Al-Mandaq.	
Bani Hasan.	
Al Mikhwah.	
Al Qara.	
Qalwa.	
Gamed zenad.	
Yes.	Were you diagnosed with diabetes before pregnancy?
No.	
Type 1 diabetes (insulin-dependent diabetes)	If the answer to the previous question is yes-What is the type of diabetes
Type 2 diabetes (non-insulin-dependent diabetes)	
1 Time.	How often are you pregnant?
2 Times.	
3 Times.	
4 Times.	
5 Times.	
6 Times.	
7 Times.	
8 Times.	
9 Times.	
10 Times.	
Yes.	Have you been diagnosed with gestational diabetes?
No.	
Less than 50 kg.	How much was your weight during pregnancy?
50 _70 kg.	
70_90 kg.	
More than 90 kg.	
Less than 37 weeks.	How long was the pregnancy?
37 to 42 weeks.	
More than 42 weeks.	
Normal delivery.	Type of delivery?
Cesarean section.	
Male.	What is the gender of the newborn?
Female.	
Less than 1 kg.	How much was the weight of the baby after the birth?
1-1.5 kg.	
1.5-2 kg.	
2-2.5 kg.	
2.5-3 kg.	
3-3.5 kg.	
3.5-4 kg.	
4-4.5 kg.	
4.5-5 kg.	
more than 5 kg.	
Yes.	Did you have high blood pressure during pregnancy (preeclampsia)?
No.	
I don’t know.	
Yes.	Did you have thyroid disease during pregnancy?
No.	
I don’t know.	
Thyroid activity.	If the answer was yes in the previous question, did you get hurt?
Thyroid inactivity.	
I don’t know.	
Yes.	Did you have bleeding during pregnancy?
No.	
I don’t know.	
Yes.	Did you have bleeding after giving birth?
No.	
I don’t know.	
Yes.	Did you have vaginal infections during pregnancy (vaginal fungi)?
No.	
I don’t know.	
Yes.	Did you have type 2 diabetes after pregnancy?
No.	
I don’t know.	
Yes.	Has the fetus been aborted?
No.	
Yes.	Increased amniotic fluid around the fetus?
No.	
I don’t know.	
Yes.	Difficult during delivery?
No.	
I don’t know.	
Yes.	Did the baby cry immediately after giving birth?
No.	
I don’t know.	
Yes.	Convulsions of the newborn after birth?
No.	
I don’t know.	
Yes.	Has the newborn intensive care been introduced? If yes what the cause
No.	
I don’t know.	
Yes.	Did the baby have jaundice (yellowing of the skin)?
No	
I don’t know.	
Yes.	Did the baby suffer from respiratory distress/lack of oxygen after birth?
No.	
I don’t know.	
Yes.	Did the baby have a deficiency in blood sugar?
No.	
I don’t know.	
